# Supplementary material for: Examining the Implementation of the Performance-Based Financing Equity Strategy in Improving Access and Utilization of Maternal Health Services in Cameroon: A Qualitative Study
Source: Int J Environ Res Public Health. 2022 Oct 29;19(21):14132. doi: 10.3390/ijerph192114132 (PMC9653716; doi:10.3390/ijerph192114132)
Supplement: Supplementary file 1 [file ijerph-19-14132-s001.zip › ijerph-1945881-supplementary.pdf]

International Journal of
Environmental Research and Public Health

MDPI

Supplementary Table S1. Using the PROGRESS lens by O'Neil et al. on the PBF design equity elements and considerations for poor and vulnerable.

PROGRESS Elements [17]	Explanation in PBF Context	Actors in the Identification/Validation Process of PAV	Level of Assessment at Health Facility	Individual/Community and Contextual Factors
Regional location of districts and place of residence	Consideration of regional and geographic barriers and population density in the allocation of equity bonuses for health facilities and districts which also has a potential trickle-down effect at the individual level especially for the poor and vulnerable (PAV)	Community health workers (CHW), quarters heads and General Supervisor at health facility	Health facility or social service department assess referred patient to qualify as PAV	CHW explore and identify potential PAV women from the health areas and validate with quarter heads for referral to health facility
Cultural sensitivity	Consideration of certain cultural groups of women not using health facility and language	Traditional birth attendants & CHW refer women to health facilities	Individual and health facility	Traditional birth attendants are encouraged to refer women to the health facility for skilled birth delivery
Employment characteristics and status	Consideration of the nature of the job and employment status of individuals	General supervisor of health facility or social service department /CHW	Health facility or social service department assess occupation status in the validation process of poor and vulnerable	CHW explore potential occupation status during identification and referral process
Gender group	Gender consideration especially for women	Health facilities/CHW	Health facility assess pregnant women based on marital status and consideration of HIV status	Women who are single or widowed are potential beneficiaries in the referral process of CHW to health facility as a potential poor and vulnerable
Religious background	All confessional health sectors are considered in the context of PBF for poor and vulnerable and all individual religious background	Not applicable	Not applicable	Not applicable
Level of education	Consideration of educational level of individuals in identifying poor and vulnerable at the health facility	General supervisor of health facility or social service department /CHW	Health facility conduct assessment of educational status during validation process	CHW explore educational status in the identification and referral process
Economic and income status	Consideration of income levels and social status of individuals for consideration as poor and vulnerable	General supervisor of health facility or social service department /CHW	Health facility or social service department conduct assessment during validation process	CHW explore income status in the process of identification and referral based on conditions of the household and understanding of the household within the health area and validates

				with quarter head for referral
Social network and family	Consideration of social network or family relatives, friends or neighbours of goodwill who can support poor and vulnerable individuals)	General supervisor of health facility or social service department /CHW	Health facility or social service department assess potential family relatives who can pay for services in the validation process	CHW explore potential social networks and family relation who can support payment and validates with quarter head for referral

Source: Adapted from O'Neil et al., 2013[17]. The first column is based on O'Neil et al's PROGRESS lens elements and the remaining four columns is based on the authors' application in the context.
